# Diagnostic utility of hematological and biochemical markers for cystic echinococcosis in Tibetan patients of Sichuan, China

**DOI:** 10.3389/fcimb.2025.1615007

**Published:** 2025-08-20

**Authors:** Meng Ma, Hao Yan, Liang Shen, Chongwei Zhang, Juan Long

**Affiliations:** ^1^ Department of Laboratory Medicine and Sichuan Provincial Key Laboratory for Human Disease Gene Study, Sichuan Provincial People’s Hospital, School of Medicine, University of Electronic Science and Technology of China, Chengdu, Sichuan, China; ^2^ Department of Laboratory Medicine, Ganzi Tibetan Autonomous Prefecture People’s Hospital, Kangding, Sichuan, China; ^3^ Department of Laboratory Medicine, West China Hospital, Sichuan University, Chengdu, Sichuan, China

**Keywords:** cystic echinococcosis, hematological marker, biochemical markers, laboratory diagnosis, plasminogen time

## Abstract

**Objective:**

This hospital-based case-control study aims to evaluate hematological and biochemical markers for the diagnosis of cystic echinococcosis (CE) in the Tibetan population of Sichuan.

**Methods:**

This study involved 83 patients diagnosed with CE and 45 healthy controls. Diagnosis of CE was confirmed through antibody and imaging tests, followed by an analysis of differences in blood and biochemical markers.

**Results:**

(1) Patients with CE displayed significant abnormalities in blood and biochemical indicators compared to healthy subjects, including increased levels of platelet count, eosinophil percentage, basophil percentage, prothrombin time, fibrinogen, activated partial thromboplastin time, total bilirubin, direct bilirubin, γ-glutamyltransferase, aspartate aminotransferase, and alkaline phosphatase. Conversely, there was a decrease in lymphocyte percentage, hemoglobin concentration, mean corpuscular volume, and hematocrit. (2) Prothrombin time levels were markedly elevated beyond normal reference values, with prolonged prothrombin time identified as a significant predictor for CE. (3) The area under the receiver operating characteristic curve (AUC) for predicting CE based on prothrombin time was 0.969, while the AUC for predicting CE using a combination of prothrombin time and eosinophil percentage was 0.982.

**Conclusion:**

Prolonged prothrombin time serves as a crucial indicator for CE, and its combination with eosinophil percentage significantly improves diagnostic accuracy, offering a potentially useful screening strategy in resource-limited endemic regions.

## 1 Introduction

Zoonotic parasitic diseases are infectious parasitic infections that are transmitted from vertebrates, especially livestock and wildlife, to humans. These diseases pose a considerable public health threat, with significant repercussions in multiple areas, including public health, livestock production, and ecological stability. They are a critical area of focus in the prevention and management of global infectious diseases (Kolören and Dubey, 2020; Javed and Alkheraije, 2023). Certain pathogens can cause severe organ damage and even death, such as *Cryptosporidiosis*, *Toxoplasma gondii* and *Echinococcus granulosus*, imposing an especially severe burden on underdeveloped countries with limited health infrastructure (Tenter et al., 2001; Ramirez et al., 2004; Malik et al., 2024). These diseases have resulted in considerable economic setbacks for the global livestock sector, leading to reduced productivity and interruptions in supply chains. Additionally, they disturb the ecological balance between parasites and their hosts. Furthermore, the excessive use of antiparasitic drugs may potentially contribute to the spread of resistance (Ryan et al., 2016).

Cystic echinococcosis (CE) is a prevalent zoonotic parasitic disease resulting from the larval stage of *Echinococcus granulosus* (*E. granulosus*). It is estimated that there are around 200,000 new cases globally each year, which poses a considerable public health concern, particularly in pastoral areas (Xiao et al., 2013; Borhani et al., 2024; Hogea et al., 2024). The Ganze Tibetan Autonomous Prefecture (30.05°N, 101.96°E), a high-altitude (avg. 3,500m) region in western Sichuan Province, China, sustains a predominantly Tibetan pastoral population of ~1.1 million. This area has emerged as a hyperendemic focus for CE due to three synergistic factors: (1) high-altitude pastoral ecosystems that perpetuate *E. granulosus* transmission cycles between livestock and domestic dogs; (2) cultural practices such as domestic slaughtering and the ritualistic disposal of contaminated offal contribute to ongoing environmental pollution; (3) systemic challenges in veterinary service coverage and public health infrastructure that limit zoonotic disease control. These determinants collectively sustain one of China’s highest CE burdens, with reported community prevalences exceeding 10% in adjacent Tibetan areas (Wang et al., 2014; Alvi et al., 2021; Hua et al., 2022).

The ongoing endemicity of CE in Tibetan communities poses a significant threat to the health of local herders and exacerbates clinical outcomes due to delays in diagnosis (Fu et al., 2021; Xu et al., 2025). While current diagnostic standards require multimodal integration of (1) epidemiological risk assessment; (2) clinical evaluation of organ-specific manifestations; (3) imaging confirmation (ultrasonography following WHO-IWGE classification and CT/MRI); and (4) serological testing (IgG ELISA with immunoblotting confirmation), resource limitations in underserved regions critically constrain implementation (Santucciu et al., 2020; Tamarozzi et al., 2021). In these settings, blood biochemical and serological tests are essential for early detection and monitoring of the disease, primarily due to their non-invasive nature, cost-effectiveness, and accessibility. Nevertheless, several challenges hinder the effectiveness of traditional serological assays, including intricate immune responses characterized by dynamic antibody profiles and antigenic diversity, high prevalence of co-infections with other parasites such as alveolar CE and cysticercosis, and logistical difficulties in sample collection and preservation in resource-poor conditions, which critically affect the practicality of these tests (Wang et al., 2008; Li et al., 2020).

The majority of current diagnostic studies on CE have primarily concentrated on regions with improved sanitation, with limited attention given to the distinct challenges posed pastoral ecosystems in economically disadvantaged settings (Sayek et al., 2004; Tao et al., 2024). This study systematically evaluates serological and biochemical markers in CE patients from the Ganze Prefecture, aiming to (1) assess the diagnostic performance of key laboratory indicators; and (2) develop an optimized, cost-effective multi-parameter diagnostic framework tailored to resource-limited regions (Rinaldi et al., 2014; Tamarozzi et al., 2014). By integrating region-specific epidemiological and clinical data, our findings could improve early CE detection and inform targeted control strategies in similar endemic zones (Nunnari et al., 2012; Govindasamy et al., 2023).

## 2 Methods

### 2.1 Diagnosis and data collection in cystic echinococcosis

This case-control study retrospectively analyzed 83 CE patients and 45 healthy controls from the People’s Hospital of Ganze Tibetan Autonomous Prefecture (2020-2022). CE cases were diagnosed based on seropositivity for anti-*E. granulosus* lgG and characteristic CT findings (unilocular/multilocular hepatic cysts with pathognomonic signs), while controls were age/sex-matched healthy volunteers with negative serology, normal imaging, and no parasitic history. Exclusion criteria for both groups included active hepatic disease, anticoagulant use, pregnancy, or malignancy. The study was approved by the institutional ethics committee, with all participants providing informed consent for anonymized data use.

### 2.2 Laboratory indexes detection

Fasting venous blood (2 mL) was collected early in the morning, and indexes were analyzed using automatic blood cell analyzer (BC-7500CRP, Mindray), automatic blood coagulation analyzer (CS-2500, Sysmex) and automatic biochemical analyzer (Cobas8000, Roche). The indicators of routine blood tests included the white blood cell count (WBC), neutrophil percentage (NEU%), lymphocyte percentage (LYM%), monocyte percentage (MON%), eosinophil percentage (EOS%), basophils percentage (BAS%), red blood cell count (RBC), hemoglobin concentration (HGB), hematocrit (HCT), mean corpuscular volume (MCV), mean corpuscular hemoglobin content (MCH), platelet count (PLT). Routine coagulation indicators included plasminogen time (PT), activated partial thromboplastin time (APTT), fibrinogen (FIB), and prothrombin time (TT). Blood biochemical indicators included total bilirubin (TBIL), direct bilirubin (DBIL), indirect bilirubin (IBIL), aspartate aminotransferase (AST), alanine aminotransferase (ALT), gamma-glutamyl transferase (GGT), alkaline phosphatase (ALP). The abnormalities in routine blood tests and blood biochemical indices were judged by clinical application standards. The reference range and clinical significance of the indicators are shown in **Supplementary Table S1**.

### 2.3 Statistical analysis

Statistical analysis was performed using Jamovi and SPSS. Normally distributed measurement data were expressed as mean ± standard deviation ( $\bar{X}$ ± S ), while count data were presented as numbers (percentage). An independent samples t-test was used to compare the differences in indicators between patients and healthy controls; the χ² test was employed to compare the abnormal rates of indicators between the two groups. For logistic regression analysis of the influencing factors of CE: First, single-variable regression analysis was conducted to screen statistically significant indicators associated with CE. These indicators were further refined through correlation analysis and collinearity diagnostics to ensure that the absolute correlation coefficients between selected variables were below 0.7 and the variance inflation factors were less than 10, thus avoiding multicollinearity. Finally, multivariate regression analysis was performed using the filtered indicators as covariates, adopting a forward stepwise selection method. The diagnostic value of CE was evaluated using the receiver operating characteristic (ROC) curve. Additionally, the predicted probability logit(p) from the logistic regression model was used as an independent variable to construct a multi-indicator combined ROC curve. P-value < 0.05 was considered statistically significant.

## 3 Results

### 3.1 Demographic and clinical characteristics

The demographic and clinical characteristics of the study participants are presented in **Supplementary Table S2**. The cystic echinococcosis (CE) patient group (n=83) and healthy controls (n=45) showed comparable age distributions and gender proportions. However, CE patients reported significantly higher exposure to livestock, highlighting this as a key risk factor for infection. Among CE cases, 31.3% demonstrated multi-organ involvement on imaging, indicating advanced disease progression in nearly one-third of patients.

### 3.2 Abnormal rates of blood and biochemical indexes in in CE patients

**Figure 1:**
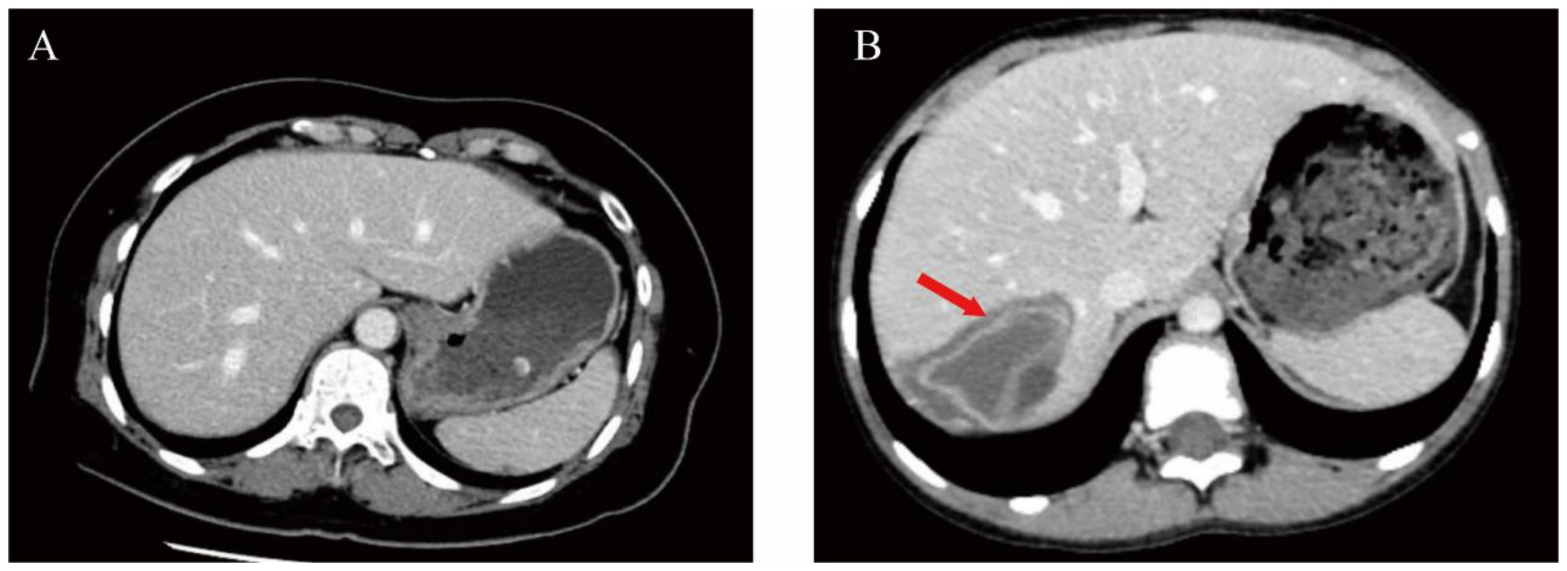
Comparative abdominal CT imaging features in healthy individuals versus patients with hepatic CE. **(A)** Abdominal CT in normal subjects. **(B)** A patient with CE infection (arrowhead illustrate the pathological pattern associated with the collapse).

A conclusive diagnosis of CE was established in 83 patients using a dual-modality diagnostic strategy that combined serum anti-*Echinococcus granulosus* IgG detection via ELISA and distinctive CT imaging characteristics. Representative CT features, including hepatic cyst localization and typical morphological patterns, are illustrated in **Figure 1**.

A retrospective analysis of laboratory parameters in CE patients versus healthy controls revealed significant abnormalities in multiple hematological and biochemical markers. Many values fell outside the normal range, with notably high abnormality rates for red blood cell (RBC) counts (61.45%), hemoglobin (HGB; 63.86%), hematocrit (HCT; 65.06%), prothrombin time (PT; 72.29%), and gamma-glutamyl transferase (GGT; 55.42%) (**Table 1**).

**Table 1:** Comparison of abnormal laboratory index rates between normal individuals and patients.

Indicator	Abnormal rate of indicators (%)		χ2	P
	Normal	Patients		
WBC (10^9^/L)	8.89%	16.87%	1.537	0.2151
RBC (10^12^/L)	51.11%	61.45%	1.278	0.2583
PLT (10^9^/L)	4.44%	28.92%	10.05	<0.01
HGB (g/L)	62.22%	63.86%	0.0335	0.8548
HCT (%)	60.53%	65.06%	0.2316	0.6304
MCV (fL)	17.78%	35.62%	4.319	<0.05
NEU% (%)	4.44%	18.07%	4.705	<0.05
LYM% (%)	11.11%	43.84%	13.85	<0.01
MON% (%)	2.22%	4.82%	0.5243	0.4690
EOS% (%)	2.22%	25.30%	10.92	<0.01
BAS% (%)	8.89%	34.25%	9.657	<0.01
PT (s)	2.5%	72.29%	52.59	<0.01
APTT (s)	12.50%	30.12%	4.544	<0.05
TT (s)	0.00%	2.41%	0.9798	0.9898
FIB (g/L)	15.00%	44.58%	10.39	<0.01
AST (U/L)	26.67%	34.94%	0.9173	0.3382
ALP (U/L)	10.00%	50.60%	19.01	<0.01
GGT(U/L)	31.11%	55.42%	6.925	<0.01
TBIL (umol/L)	0.00%	15.66%	7.845	<0.01
DBIL (umol/L)	4.44%	28.92%	10.8	<0.01
IBIL (umol/L)	0.00%	12.05%	5.881	<0.05

### 3.3 Comparison of laboratory indexes between CE patients and control group

Compared to healthy controls, CE patients exhibited significantly elevated platelet counts (PLT), eosinophil (EOS%) and basophil (BAS%) percentages, PT, activated partial thromboplastin time (APTT), fibrinogen (FIB), total bilirubin (TBIL), direct bilirubin (DBIL), GGT, aspartate aminotransferase (AST), and alkaline phosphatase (ALP). Conversely, HGB, lymphocyte percentage (LYM%), mean corpuscular volume (MCV), and HCT were significantly lower (**Table 2**).

**Table 2:** Comparison of laboratory indexes between normal individuals and patients.

Indicator	Controls (x ± s)	CE Patients (x ± s)	P
PLT (10^9^/L)	224 ± 48.4	270 ± 76.1	<0.01
HGB (g/L)	149 ± 27.5	135 ± 28.5	<0.01
HCT (%)	47.3 ± 7.4	41.3 ± 8.09	<0.01
MCV (fL)	95.5 ± 7.45	84.4 ± 7.83	<0.01
LYM% (%)	29.7 ± 7.5	24.3 ± 9.81	<0.01
EOS% (%)	2.49 ± 1.62	6.24 ± 5.94	<0.01
BAS% (%)	0.636 ± 0.349	0.810 ± 0.515	<0.05
APTT (s)	26.9 ± 2.66	32.3 ± 12.4	<0.01
PT (s)	11.1 ± 0.662	15.1 ± 7.58	<0.05
FIB (g/L)	2.84 ± 0.852	3.29 ± 1.15	<0.05
TBIL (umol/L)	10.5 ± 3.56	32.2 ± 68.3	<0.05
DBIL (umol/L)	4.17 ± 1.58	22.1 ± 53.4	<0.05
AST (U/L)	22.8 ± 11.2	37.3 ± 42.4	<0.05
ALP (U/L)	87.7 ± 40.1	204 ± 278	<0.01
GGT (U/L)	60.8 ± 72.8	125 ± 178	<0.05

Abnormality rate analysis further demonstrated that PLT, MCV, neutrophil percentage (NEU%), LYM%, EOS%, BAS%, PT, APTT, FIB, TBIL, DBIL, indirect bilirubin (IBIL), and GGT were significantly more frequent in CE patients. Other indices showed no statistically significant differences between groups (**Table 1**).

### 3.4 Correlation analysis of blood and biochemical indexes with CE

The results of one-way regression analysis of the hematological and biochemical markers of CE showed that PLT, EOS%, BAS%, PT, APTT, FIB, GGT, AST, and ALP were the risk factors for CE, as shown in **Table 3**. These indexes were chosen as covariates for the logistic step-by-step method of analysis, the results showed that the risk of CE increased with the levels of PT, as shown in **Table 4**.

**Table 3:** Single factor regression analysis of risk factors for CE.

Indicator	Estimate	SE	Wald	OR	95%CI	P
PLT (10^9^/L)	0.0106	0.00322	10.888	1.011	1.0043-1.017	<0.001
EOS% (%)	0.321	0.0872	13.537	1.379	1.162-1.64	<0.001
BAS% (%)	0.8771	0.444	3.895	2.404	1.006-5.74	0.048
APTT (s)	0.218	0.068	1.329	1.244	1.089-1.422	0.001
PT (s)	3.434	0.689	4.982	31.004	8.029-119.724	<0.001
FIB (g/L)	0.419	0.207	4.107	1.521	1.014-2.281	0.043
GGT (U/L)	0.00605	0.00286	4.473	1.01	1-1.01	0.034
AST (U/L)	0.0241	0.0123	3.875	1.024	1-1.05	0.049
ALP (U/L)	0.0118	0.00446	6.987	1.012	1.003	0.008

**Table 4:** Multivariate regression analysis of risk factors for CE.

Indicator	Estimate	SE	Wald	OR	95%CI	P
PT (s)	3.942	1.082	13.281	51.527	6.184-429.343	<0.001
Intercept	-55.714	15.205	13.426			

### 3.5 Diagnostic performance evaluation by ROC analysis

**Figure 2:**
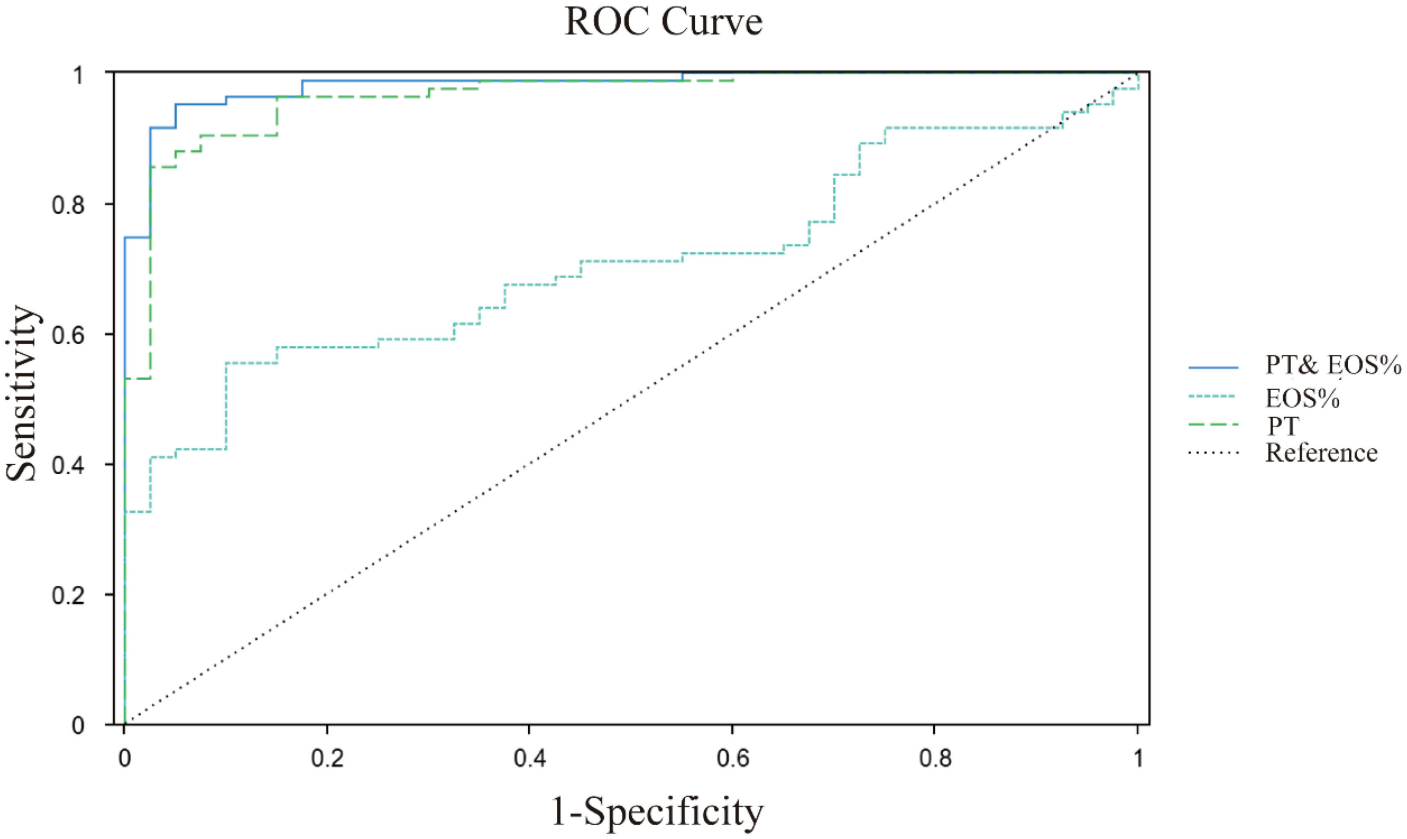
ROC curves analysis of PT, EOS%, PT&EOS% for CE prediction. PT (green dashed line): cutoff >12.2s, AUC 0.969; EOS% (blue dashed line): cutoff >4.2, AUC 0.720; PT and EOS% (blue solid line): AUC 0.982; Diagonal grey line represents reference for random prediction. All curves show statistically significant discrimination (p<0.001).

The ROC curve analysis revealed significant differences in diagnostic capabilities among the evaluated indices (**Figure 2**). PT demonstrated exceptional accuracy, achieving an AUC of 0.969 (95% CI: 0.940 ~ 0.997), whereas EOS% displayed moderate predictive value with an AUC of 0.720 (95% CI: 0.634 ~ 0.806). In contrast, other markers, including PLT, BAS%, APTT, FIB, GGT, AST, and ALP, exhibited limited diagnostic utility, with AUC values below 0.7. Notably, the combination of PT and EOS% achieved superior performance (AUC = 0.982, 95% CI: 0.902~1.001), indicating synergistic diagnostic value for CE (**Table 5**).

**Table 5:** The predictive value of PT, EOS%, PT and EOS% analysis for CE.

Indicator	AUC	95%CI	Cut off	Sensitivity	Specificity	P
PT (s)	0.969	0.940 ~ 0.997	12.2	0.855	0.975	<0.001
EOS% (%)	0.72	0.634 ~ 0.806	4.2	0.554	0.911	<0.001
PT and EOS%	0.982	0.902~1.001	–	0.952	0.950	<0.001

## 4 Discussion

This research investigated the relationship between hematological and biochemical parameters in the Tibetan population and cystic echinococcosis (CE), uncovering significant laboratory indicators that offer essential data for the prevention and diagnosis of cystic echinococcosis. CE can induce abnormalities in routine blood and biochemical indicators. Hematological analysis revealed increased abnormality rates in red blood cells (RBC), hemoglobin (HGB), hematocrit (HCT), and mean corpuscular volume (MCV), with all these parameters showing significantly lower concentrations compared to the normal control group. These findings collectively reflect reduced hemoglobin levels, suggesting impaired oxygen-carrying capacity (Klisic et al., 2023; Wen et al., 2024). The simultaneous decline in these markers may indicate chronic inflammation in CE patients, leading to accelerated erythrocyte destruction, compromised oxygen transport, and subsequent tissue hypoxia, which could result in neurological dysfunction or severe respiratory/circulatory impairment. Alternatively, these abnormalities may signal anemia, warranting clinical vigilance.

Thrombocytosis, another observed abnormality, poses additional health risks by potentially elevating thrombotic susceptibility, as platelets primarily function in hemostasis and thrombosis prevention (Teodorico et al., 2024). However, excessive platelet (PLT) counts may promote unwanted intravascular clot formation, increasing the risk of thrombotic disorders such as myocardial infarction or stroke. While CE itself already compromises patient health, concomitant thrombocytosis could further exacerbate these risks. For instance, CE-induced liver or multi-organ damage may synergize with elevated PLT to heighten susceptibility to hemorrhage or thrombosis (Fei et al., 2024).

Serological analyses also revealed significant elevations in EOS% and BAS% among CE patients. Both cell types are pivotal in immune responses, particularly against parasitic infections and allergic reactions (Wang et al., 2023). Their elevation in echinococcal infection suggests immune hyperactivation (Liu et al., 2022). While this exhibits a protective effect, excessive quantities may lead to tissue damage or other complications. ROC curve analysis indicated strong predictive utility for EOS% in CE diagnosis (AUC = 0.72; optimal cutoff: 4.2%). Collectively, these serological aberrations reflect both host anti-parasitic responses and a chronic inflammatory state, which may predispose patients to thrombosis, nutritional deficits, and systemic health decline. Thus, comprehensive CE management should extend beyond primary treatment to include vigilant monitoring and correction of these hematological derangements to optimize clinical outcomes.

Total bilirubin (TBIL), direct bilirubin (DBIL), gamma-glutamyl transferase (GGT), aspartate aminotransferase (AST), and alkaline phosphatase (ALP) levels are significantly elevated in patients with CE. The significant increase in TBIL, DBIL, and GGT is attributed to the compressive impact of intrahepatic cysts on the bile ducts (Miletic et al., 2024). Elevated levels of GGT and ALP (established markers of cholestasis), along with AST (a marker of hepatocellular injury) provide evidence of compromised liver function. The changes in these secretions and enzymes may be caused by the direct invasion of parasites into liver tissue or by secondary complications. Interestingly, these indicators showed a more pronounced increase in patients with concurrent infections (**Supplementary Table S3**), suggesting that the presence of concurrent infections exacerbates liver function deterioration.

Notably, we observed significant prolongation of prothrombin time (PT) in CE patients (AUC = 0.969, optimal cutoff >12.2 s). While no direct causal relationship between CE and PT prolongation has been established, our data suggest that this phenomenon reflects secondary hepatic dysfunction, as 91% of affected cases showed imaging-confirmed liver involvement, with the majority concurrently exhibiting abnormal liver enzymes. The mechanistic basis may involve impaired synthesis of vitamin K-dependent coagulation factors (II, VII, IX, and X) due to cystic lesions and associated inflammation, which compromises hepatic synthetic function (Tao et al., 2022). This hypothesis is supported by the strong correlation between PT prolongation and other hepatic injury markers.

The combination of prothrombin time (PT) and eosinophil percentage (EOS%) showed enhanced diagnostic accuracy (AUC = 0.982) in clinical settings, indicating that these biomarkers represent distinct pathological processes: PT reflects liver dysfunction, while EOS% indicates an immune response to parasitic infection. These results suggest that coagulation testing may serve as a useful supplementary diagnostic tool in regions endemic for CE.

Recent studies have indicated a relationship between blood biomarkers and CE. For instance, a study from Iran found that parasite-derived miRNAs, such as egr-miR-71, can be detected early during infection (Habibi et al., 2023). Meanwhile, research from Austria indicated that eosinophil-derived protein (ECP) levels are significantly elevated in populations infected with CE, which aligns with our perspective (Hotz et al., 2022). Additionally, a cohort study conducted in Italy and Turkey identified immune regulatory factors Src and Lyn as being associated with the activity of CE (Fratini et al., 2020). Although there are differences in biomarkers across various regions, common pathophysiological characteristics, chronic inflammation, liver dysfunction, and parasite immune evasion responses are observed following CE infection. The aforementioned biomarker detection methods are complex, costly, and not readily available in routine clinical practice. In contrast, our approach utilizes easily accessible routine hematological parameters (EOS%/PT), making it highly practical in resource-limited settings (Khan et al., 2023; Al-khlifeh et al., 2024).

As a paradigm of zoonotic parasitic disease, cystic echinococcosis (CE) highlights the complex interdependencies among animal reservoirs, environmental determinants, and human infection. The implementation of early diagnostic protocols and targeted prevention strategies against CE carries substantial scientific and public health significance (Abbas et al., 2020; Dubey, 2021; Almuzaini, 2023). Integrating specific biomarkers into existing CE screening and monitoring programs holds promise for significantly enhancing early detection and risk stratification in public health efforts. For example, biomarkers such as EOS% (with an AUC of 0.72) and PT (with an AUC of 0.969) demonstrate strong predictive value for CE diagnosis, respectively, positioning them as cost-effective tools for resource-constrained settings. Moreover, population-level biomarker data can strengthen burden-of-disease assessments, guiding prioritization of resource allocation in endemic regions. Integrating these established biomarkers into the continuous monitoring system can facilitate early prevention, detection, and treatment, ultimately minimizing the health risks and socio-economic consequences associated with zoonotic diseases.

## 5 Conclusion

This study identifies basic hematological parameters, specifically prolonged prothrombin time (PT >12.2s) combined with eosinophil percentage, as highly accurate diagnostic biomarkers for cystic echinococcosis (AUC 0.982) in Tibetan pastoral populations. These findings address a critical diagnostic gap in resource-limited endemic areas by demonstrating that routine blood tests can effectively screen for CE where imaging technologies are inaccessible. Although the hospital-based design may affect generalizability, these cost-effective biomarkers provide a practical solution for early CE detection and could significantly enhance diagnostic capacity in comparable endemic regions. Further validation through community-based screening programs is warranted, along with investigation of their potential for monitoring treatment response and disease progression.

## Data Availability

The original contributions presented in the study are included in the article/**Supplementary Material**. Further inquiries can be directed to the corresponding authors.

## References

[B1] Abbas I. E. Villena I. Dubey J. P. (2020). A review on toxoplasmosis in humans and animals from Egypt. PARASITOLOGY 147, 135–159. doi: 10.1017/S0031182019001367, PMID: 31559938 PMC10317695

[B2] Al-khlifeh E. Alshammari A. Alnasarat H. (2024). High Incidence of G1 Genotype Found in the Levant Revealed by Sequence-based Association Analysis of Echinococcus granulosus (sensu stricto). Pakistan VETERINARY J. 44, 405–413. doi: 10.29261/pakvetj/2024.143

[B3] Almuzaini A. M. (2023). Flow of zoonotic toxoplasmosis in food chain. Pakistan VETERINARY J. 43, 1–16. doi: 10.29261/pakvetj/2023.010

[B4] Alvi M. A. Li L. Saqib M. Ohiolei J. A. Younas M. W. Tayyab M. H.. (2021). Serologic evidence of Echinococcus granulosus in slaughterhouses in Pakistan: global alarm for butchers in developing countries. J. Infect. Dev. Ctries 15, 861–869. doi: 10.3855/jidc.14029, PMID: 34242198

[B5] Borhani M. Fathi S. Harandi M. F. Casulli A. Ding J. Liu M. Y.. (2024). *Echinococcus granulosus* sensu lato control measures: a specific focus on vaccines for both definitive and intermediate hosts. Parasites Vectors 17, 533. doi: 10.1186/s13071-024-06581-2, PMID: 39716337 PMC11665232

[B6] Dubey J. P. (2021). Outbreaks of clinical toxoplasmosis in humans: five decades of personal experience, perspectives and lessons learned. Parasites Vectors 14 (4), 263. doi: 10.1186/s13071-021-04769-4, PMID: 34011387 PMC8136135

[B7] Fei Y. Xiong Z. G. Huang L. Zhang C. (2024). Construction of platelet count-optical method reflex test rules using Micro-RBC, Macro-RBC%, “PLT clumps?” flag, and “PLT abnormal histogram” flag on the Mindray BC-6800plus hematology analyzer in clinical practice. Clin. Chem. Lab. Med. 63, 329–337. doi: 10.1515/cclm-2024-0739, PMID: 39217753

[B8] Fratini F. Tamarozzi F. Macchia G. Bertuccini L. Mariconti M. Birago C.. (2020). Proteomic analysis of plasma exosomes from Cystic Echinococcosis patients provides *in vivo* support for distinct immune response profiles in active vs inactive infection and suggests potential biomarkers. PloS Negl. Trop. Dis. 14, e0008586. doi: 10.1371/journal.pntd.0008586, PMID: 33017416 PMC7535053

[B9] Fu M. H. Wang X. Han S. Guan Y. Y. Bergquist R. Abcde W. P. W. (2021). Advances in research on echinococcoses epidemiology in China. Acta TROPICA 219, 105921. doi: 10.1016/j.actatropica.2021.105921, PMID: 33878307

[B10] Govindasamy A. Bhattarai P. R. John J. (2023). Liver cystic echinococcosis: a parasitic review. Ther. Adv. Infect. Dis. 10, 20499361231171478. doi: 10.1177/20499361231171478, PMID: 37197609 PMC10184195

[B11] Habibi B. Gholami S. Bagheri A. Fakhar M. Moradi A. Khazeei Tabari M. A. (2023). Cystic echinococcosis microRNAs as potential noninvasive biomarkers: current insights and upcoming perspective. Expert Rev. Mol. Diagn. 23, 885–894. doi: 10.1080/14737159.2023.2246367, PMID: 37553726

[B12] Hogea M. O. Ciomaga B. F. Muntean M. M. Muntean A. A. Popa M. I. Popa G. L. (2024). Cystic echinococcosis in the early 2020s: A review. Trop. Med. Infect. Dis. 9 (2), 36. doi: 10.3390/tropicalmed9020036, PMID: 38393125 PMC10891927

[B13] Hotz J. F. Kaczirek K. Stremitzer S. Waneck F. Auer H. Perkmann T.. (2022). Evaluation of eosinophilic cationic protein as a marker of alveolar and cystic echinococcosis. Pathogens 11 (3), 261. doi: 10.3390/pathogens11020261, PMID: 35215203 PMC8878807

[B14] Hua R. Q. Du X. D. He X. Gu X. B. Xie Y. He R.. (2022). Genetic diversity of *Echinococcus granulosus* sensu lato in China: Epidemiological studies and systematic review. TRANSBOUNDARY AND EMERGING Dis. 69, E1382–E1392. doi: 10.1111/tbed.14469, PMID: 35139582

[B15] Javed K. Alkheraije K. A. (2023). Cryptosporidiosis: A foodborne zoonotic disease of farm animals and humans. Pakistan VETERINARY J. 43, 213–223. doi: 10.29261/pakvetj/2023.038

[B16] Khan S. Younus M. Cable J. Hailer F. Idrees A. Rashid M. I.. (2023). Epidemiology of bovine hydatidosis: urbanization, dogs, animal care and proximity to slaughterhouses are important risk factors for cattle. Pakistan VETERINARY J. 43, 507–514. doi: 10.29261/pakvetj/2023.055

[B17] Klisic A. Vujacic I. R. Kostadinovic J. Ninic A. (2023). Red cell distribution width is inversely associated with body mass index in late adolescents. Eur. Rev. FOR Med. AND Pharmacol. Sci. 27, 7148–7154. doi: 10.26355/eurrev_202308_33288, PMID: 37606125

[B18] Kolören Z. Dubey J. P. (2020). A review of toxoplasmosis in humans and animals in Turkey. PARASITOLOGY 147, 12–28. doi: 10.1017/S0031182019001318, PMID: 31554526 PMC10317629

[B19] Li S. Chen J. He Y. Deng Y. Chen J. Fang W.. (2020). Clinical features, radiological characteristics, and outcomes of patients with intracranial alveolar echinococcosis: A case series from tibetan areas of Sichuan province, China. Front. Neurol. 11, 537565. doi: 10.3389/fneur.2020.537565, PMID: 33519658 PMC7843382

[B20] Liu Z. S. Zhang R. L. Liu Y. N. Ma R. Z. Zhang L. G. Zhao Z.. (2022). Eosinophils and basophils in severe fever with thrombocytopenia syndrome patients: Risk factors for predicting the prognosis on admission. PLoS Negl. Trop. Dis. 16 (12), e0010967. doi: 10.1371/journal.pntd.0010967, PMID: 36542604 PMC9770358

[B21] Malik M. A. Subhani M. I. Alvi M. A. Wakid M. H. Alkhaldi A. A. M. Saqib M.. (2024). High Genetic Variability in Full-length cox2 and nad6 Genes of Echinococcus granulosus sensu stricto and Echinococcus ortleppi Recovered from Cattle. Pakistan VETERINARY J. 44, 148–154. doi: 10.29261/pakvetj/2024.141

[B22] Miletic B. Sutter Y. Buset E. Segulja S. Hesse M. (2024). Icterus provoked by Echinococcus mimicking a tumor: A diagnostic challenge. Travel Med. Infect. Dis. 63, 102790. doi: 10.1016/j.tmaid.2024.102790, PMID: 39617151

[B23] Nunnari G. Pinzone M. R. Gruttadauria S. Celesia B. M. Madeddu G. Malaguarnera G.. (2012). Hepatic echinococcosis: Clinical and therapeutic aspects. World J. OF Gastroenterol. 18, 1448–1458. doi: 10.3748/wjg.v18.i13.1448, PMID: 22509076 PMC3319940

[B24] Ramirez N. E. Ward L. A. Sreevatsan S. (2004). A review of the biology and epidemiology of cryptosporidiosis in humans and animals. Microbes AND INFECTION 6, 773–785. doi: 10.1016/j.micinf.2004.02.021, PMID: 15207825

[B25] Rinaldi F. Brunetti E. Neumayr A. Maestri M. Goblirsch S. Tamarozzi F. (2014). Cystic echinococcosis of the liver: A primer for hepatologists. World J. Hepatol. 6, 293–305. doi: 10.4254/wjh.v6.i5.293, PMID: 24868323 PMC4033287

[B26] Ryan U. Zahedi A. Paparini A. (2016). Cryptosporidium in humans and animalsa one health approach to prophylaxis. PARASITE Immunol. 38, 535–547. doi: 10.1111/pim.12350, PMID: 27454991

[B27] Santucciu C. Bonelli P. Peruzzu A. Fancellu A. Marras V. Carta A.. (2020). Cystic echinococcosis: clinical, immunological, and biomolecular evaluation of patients from Sardinia (Italy). Pathogens 9 (11), 907. doi: 10.3390/pathogens9110907, PMID: 33143032 PMC7693143

[B28] Sayek I. Tirnaksiz M. B. Dogan R. (2004). Cystic hydatid disease: Current trends in diagnosis and management. Surg. Today 34, 987–996. doi: 10.1007/s00595-004-2830-5, PMID: 15580379

[B29] Tamarozzi F. Nicoletti G. J. Neumayr A. Brunetti E. (2014). Acceptance of standardized ultrasound classification, use of albendazole, and long-term follow-up in clinical management of cystic echinococcosis: a systematic review. Curr. Opin. IN Infect. Dis. 27, 425–431. doi: 10.1097/QCO.0000000000000093, PMID: 25101556

[B30] Tamarozzi F. Silva R. Fittipaldo V. A. Buonfrate D. Gottstein B. Siles-Lucas M. (2021). Serology for the diagnosis of human hepatic cystic echinococcosis and its relation with cyst staging: A systematic review of the literature with meta-analysis. PloS Negl. Trop. Dis. 15, e0009370. doi: 10.1371/journal.pntd.0009370, PMID: 33909640 PMC8081258

[B31] Tao J. Du X. Liu K. Wang C. Lv Y. Wang M.. (2022). Clinical characteristics and antibodies against Echinococcus granulosus recombinant antigen P29 in patients with cystic echinococcosis in China. BMC Infect. Dis. 22, 609. doi: 10.1186/s12879-022-07597-8, PMID: 35820830 PMC9275268

[B32] Tao Y. Wang Y. F. Wang J. Long S. Seyler B. C. Zhong X. F.. (2024). Pictorial review of hepatic echinococcosis: Ultrasound imaging and differential diagnosis. World J. Gastroenterol. 30 (37), 4115–4131. doi: 10.3748/wjg.v30.i37.4115, PMID: 39474399 PMC11514533

[B33] Tenter A. M. Heckeroth A. R. Weiss L. M. (2001). Toxoplasma gondii:: from animals to humans. Int. J. FOR Parasitol. 31, 217–220. doi: 10.1016/S0020-7519(01)00125-4 PMC3109627 11113252

[B34] Teodorico E. Moro F. Santoro A. Scaglione G. Infante A. Silvi C.. (2024). Retroperitoneal cyst with iliac stent involvement as primary manifestation of cystic echinococcosis. Ultrasound Obstet Gynecol. 65 (1), 128–129 doi: 10.1002/uog.27672, PMID: 38748908

[B35] Wang Q. Huang Y. Huang L. Yu W. J. He W. Zhong B.. (2014). Review of risk factors for human echinococcosis prevalence on the Qinghai-Tibet Plateau, China: a prospective for control options. Infect. Dis. Poverty 3 (1), 3. doi: 10.1186/2049-9957-3-3, PMID: 24475907 PMC3910240

[B36] Wang Z. H. Wang X. M. Liu X. Q. (2008). Echinococcosis in China, a review of the epidemiology of *Echinococcus* spp. ECOHEALTH 5, 115–126. doi: 10.1007/s10393-008-0174-0, PMID: 18787915

[B37] Wang J. W. Yang Y. J. Guo J. Yu P. Y. Wang G. K. Liu X. Y.. (2023). The tissue lymphocyte-to-eosinophil ratio predicted long-term recurrence of eosinophilic CRSwNP. Am. J. OF RHINOL. Allergy 37, 563–570. doi: 10.1177/19458924231179615, PMID: 37271971

[B38] Wen X. Wang F. Tang T. Xu B. Y. Yuan M. Y. Li Y. H.. (2024). Sex-specific association of peripheral blood cell indices and inflammatory markers with depressive symptoms in early adolescence. J. OF Affect. Disord. 362, 134–144. doi: 10.1016/j.jad.2024.06.098, PMID: 38960333

[B39] Xiao N. Yao J. W. Ding W. Giraudoux P. Craig P. S. Ito A. (2013). Priorities for research and control of cestode zoonoses in Asia. Infect. Dis. Poverty 2, 16. doi: 10.1186/2049-9957-2-16, PMID: 23915395 PMC3750256

[B40] Xu Y. Luo C. Liu J. Shen C. Ma X. (2025). Evaluation of knowledge, attitude and practice towards cystic echinococcosis among undergraduate students in China. PloS One 20, e0321399. doi: 10.1371/journal.pone.0321399, PMID: 40215254 PMC11990511

