# Supplementary material for: Diagnostic utility of hematological and biochemical markers for cystic echinococcosis in Tibetan patients of Sichuan, China
Source: Front Cell Infect Microbiol. 2025 Aug 20;15:1615007. doi: 10.3389/fcimb.2025.1615007 (PMC12405450; doi:10.3389/fcimb.2025.1615007)
Supplement: Supplementary file 1 [file DataSheet1.pdf]

Table S1 Reference ranges and clinical significance of laboratory parameters

Abbreviations	Full name	Male	Female	Clinical implications
WBC (10^9/L)	White Blood Cell	3.5-9.5	3.5-9.5	Reflects immune function, elevation suggests infection/inflammation
RBC (10^{12}/L)	Red Blood Cell	4.3-5.8	3.8-5.1	Assessment of anemia or erythrocytosis
PLT (10^9/L)	Platelet	85-303	101-320	Correlates with hemostatic function, abnormalities suggest bleeding/thrombosis risk
HGB (g/L)	Hemoglobin	130-175	115-150	Core indicators for the diagnosis and classification of anemia
HCT (%)	Hematocrit	40.0-50.0	35.0-45.0	Reflects blood concentration/dilution status
MCV (fL)	Mean Corpuscular Volume	82.0-100.0	82.0-100.0	For morphologic classification of anemia (macro/ortho/microcytic)
NEU% (%)	Neutrophil Percentage	40.0-70.0	40.0-70.0	Typically elevated in bacterial infections
LYM% (%)	Lymphocyte Percentage	20.0-50.0	20.0-50.0	Changes during viral infections/immune diseases
MON% (%)	Monocyte Percentage	3.0-10.0	3.0-10.0	Chronic inflammation/tuberculosis etc. can be elevated
EOS% (%)	Eosinophil Percentage	0.4-8.0	0.4-8.0	Elevated in allergies/parasitic infections
BAS% (%)	Basophil Percentage	0-1.0	0-1.0	Rarely elevated, seen in myeloproliferative disorders
PT (s)	Prothrombin Time	9.7-12.6	9.7-12.6	Assessment of exogenous coagulation pathways for anticoagulation monitoring
APTT (s)	Activated Partial Thromboplastin Time	23.3-32.5	23.3-32.5	Evaluation of endogenous coagulation pathways
TT (s)	Thrombin Time	14-21	14-21	Reflects abnormal fibrinogen function
FIB (g/L)	Fibrinogen	1.8-3.5	1.8-3.5	Important indicators for DIC diagnosis
AST (U/L)	Aspartate Aminotransferase	15-40	15-40	Hepatocyte/myocardial injury marker

ALT (U/L)	Alanine Aminotransferase	9-50	7-40	Elevated ALT primarily indicates hepatocellular injury
ALP (U/L)	Alkaline Phosphatase	45-125	35-100	Elevated in hepatobiliary/bone disease
GGT (U/L)	Gamma-Glutamyl Transferase	10-60	7-45	Hepatobiliary Disease-Specific Indicators
TBIL (umol/L)	Total Bilirubin	0-26	0-21	Assessing the degree of jaundice
DBIL (umol/L)	Direct Bilirubin	0-8	0-8	Elevation suggests cholestasis/biliary obstruction
IBIL (umol/L)	Indirect Bilirubin	0-20	0-20	Significantly higher in hemolytic diseases

Table S2 Demographic and Clinical Characteristics

Characteristic	CE Patients (n=83)	Controls (n=45)	P
Age (years)	43.1（±15.8）	44.5（±8.19）	0.579
Male (%)	46.98	48.89	0.848
Livestock contact	0.867	0.333	<0.001
Multi-organ involvement	31.33		

Table S3 Comparative analysis of laboratory indicators among healthy individuals, CE patients, and CE co-infection patients

Indicator	Controls (x±s)	CE Patients (x±s)	Group A (x±s)	Group B (x±s)
WBC (10^9/L)	6.01±1.48	6.82±2.58	6.92±2.59*	6.17±2.56
RBC (10^{12}/L)	4.96±0.713	4.88±0.847	4.81±0.814	5.34±0.955
PLT (10^9/L)	224±48.4	270±76.1***	278±76.2***	219±54.8[a]
HGB (g/L)	149±27.5	135±28.5***	132±28.6***	151±18.2[a]
HCT (%)	47.3±7.4	41.3±8.09***	40.5±8.18***	46.0±5.71[a]
MCV (fL)	95.5±7.45	84.4±7.83***	83.5±7.92***	90.0±4.22* [a]
NEU% (%)	60.0±8.33	62.0±12.0	62.1±12.1	61.6±11.5
LYM% (%)	29.7±7.5	24.3±9.81***	24.1±9.71***	25.4±10.9
MON% (%)	6.66±1.49	6.62±1.99	6.61±2.01	6.71±1.99
EOS% (%)	2.49±1.62	6.24±5.94***	6.35±6.06***	5.51±5.31***
BAS% (%)	0.636±0.349	0.810±0.515*	0.814±0.498*	0.782±0.643
PT (s)	11.1±0.662	15.1±7.58**	15.4±8.09***	12.9±0.792***
APTT (s)	26.9±2.66	32.3±12.4***	32.9±13.1**	28.5±4
TT (s)	17.8±0.659	18.0±1.39	18.0±1.38	18.2±1.54
FIB (g/L)	2.84±0.852	3.29±1.15*	3.47±1.16*	3.10±1.08
AST (U/L)	22.8±11.2	37.3±42.4*	33.8±37.4*	60.6±64.5***
ALT (U/L)	30.6±16.8	42.5±53.4	36.6±44.9	80.6±85.1*** [a]
ALP (U/L)	87.7±40.1	204±278***	211±289**	154±187*
GGT (U/L)	60.8±72.8	125±178*	124±181*	130±165*
TBIL (umol/L)	10.5±3.56	32.2±68.3*	33.2±70.9*	25.6±50.0*
DBIL (umol/L)	4.17±1.58	22.1±53.4*	23.1±55.9*	15.2±33.8*
IBIL (umol/L)	6.33±2.22	10.1±15.7	10.1±15.8	10.4±16.2

Group A: CE-infected patients only (n = 72); Group B: CE co-infection patients (n= 11).

*: Statistically significant vs. control group; a: Statistically significant between Group A and Group B.
